# Prevalence and risk factors of difficult-to-treat axial spondyloarthritis: Real-life evidence from the BioSTaR database

**DOI:** 10.1007/s10067-026-07926-1

**Published:** 2026-01-19

**Authors:** Hatice Bodur, Şebnem Ataman, Fatma Gül Yurdakul, Gülcan Gürer, Kenan Akgün, Lale Altan İnceoğlu, İsmihan Sunar, Özgür Akgül, İlhan Sezer, Erhan Çapkın, Aylin Rezvani, İlker Yağcı, Mehmet Tuncay Duruöz, Hasan Fatih Çay, Remzi Çevik, Feride Göğüş, Ayhan Kamanlı, Hilal Ecesoy, Tuba Güler, Nilay Şahin, Gizem Cengiz, Meltem Alkan Melikoğlu

**Affiliations:** 1 Department of Physical Medicine and Rehabilitation, Faculty of Medicine, Ankara City Hospital, Yıldırım Beyazıt University, Ankara, Turkey; 2 Division of Rheumatology, Department of Physical Medicine and Rehabilitation, Faculty of Medicine, Ankara University, Ankara, Turkey; 3 Department of Physical Medicine and Rehabilitation, Ankara City Hospital, University of Health Sciences, Ankara, Turkey; 4 Department of Physical Medicine Rehabilitation and Rheumatology, School of Medicine, Adnan Menderes University, Aydın, Turkey; 5 Department of Physical Medicine and Rehabilitation, Cerrahpaşa Medical Faculty, İstanbul University-Cerrahpaşa, Istanbul, Turkey; 6 Department of Physical Medicine Rehabilitation and Rheumatology, Faculty of Medicine, Uludağ University, Bursa, Turkey; 7 Division of Rheumatology, Department of Physical Medicine and Rehabilitation, School of Medicine, Manisa Celal Bayar University, Manisa, Turkey; 8 Division of Rheumatology, Department of Physical Medicine and Rehabilitation, School of Medicine, Akdeniz University, Antalya, Turkey; 9 Department of Physical Medicine and Rehabilitation, School of Medicine, Karadeniz Technical University, Trabzon, Turkey; 10 Department of Physical Medicine and Rehabilitation, International School of Medicine, İstanbul Medipol University, Istanbul, Turkey; 11 Department of Physical Medicine and Rehabilitation, Faculty of Medicine, Marmara University, Istanbul, Turkey; 12 Division of Rheumatology, Department of Physical Medicine and Rehabilitation, Faculty of Medicine, Marmara University, Istanbul, Turkey; 13 Department of Physical Medicine Rehabilitation and Rheumatology, Antalya Training and Research Hospital, University of Health Sciences, Antalya, Turkey; 14 Department of Physical Medicine and Rehabilitation, School of Medicine, Dicle University, Diyarbakır, Turkey; 15 Department of Physical Medicine Rehabilitation and Rheumatology, School of Medicine, Gazi University, Ankara, Turkey; 16 Division of Rheumatology, Department of Physical Medicine and Rehabilitation, Faculty of Medicine, Karamanoğlu Mehmetbey University, Karaman, Turkey; 17 Department of Physical Medicine and Rehabilitation, Faculty of Medicine, Balıkesir University, Balıkesir, Turkey; 18 Division of Rheumatology, Department of Physical Medicine and Rehabilitation, Faculty of Medicine, Erciyes University, Kayseri, Turkey; 19 Department of Physical Medicine Rehabilitation and Rheumatology, School of Medicine, Atatürk University, Erzurum, Turkey

**Keywords:** Axial spondyloarthritis, Difficult-to-treat, DMARD, Epidemiology, Spondyloarthropathy

## Abstract

**Objectives:**

This study was aimed at determining the prevalence of difficult-to-treat (D2T) axial spondyloarthritis (axSpA) and identifying main associated factors for D2T axSpA.

**Method:**

This multicenter observational cross-sectional study included axSpA patients from the BioSTaR (Biological and Targeted Synthetic Disease-Modifying Antirheumatic Drugs Registry) from February 1, 2019, to January 1, 2025. Data from 1800 axSpA patients who have previously used or are currently using at least one biologic/targeted synthetic disease-modifying antirheumatic drug were analyzed. Patient data included demographic characteristics, body mass index (BMI), marital status, smoking and alcohol use, family history of SpA, and presence of comorbidities. The parameters related to SpA such as disease duration, type of axSpA (radiographic/non-radiographic), HLA-B27 status, the presence of extra-musculoskeletal manifestations (uveitis, psoriasis, and inflammatory bowel disease), arthritis, enthesitis, and dactylitis were also recorded. Comorbidities including hypertension, cardiovascular disease, diabetes, obesity, and hyperlipidemia were recorded, and Charlson Index scores were evaluated. Maastricht Ankylosing Spondylitis Enthesitis Score (MASES) and disease activity in means of Bath Ankylosing Spondylitis Disease Activity Index (BASDAI) and Ankylosing Spondylitis Disease Activity Score with C-reactive protein (ASDAS-CRP) were also recorded. All medication history and currently used medications for axSpA and other diseases were noted. D2T and non-D2T axSpA patients were classified according to the suggested extrapolated definition.

**Results:**

Of the 1800 axSpA patients recorded in the BioSTaR database, 204 (11.3%) were classified as D2T axSpA. Data of these patients were compared to the data from 1596 non-D2T axSpA patients. Disease duration was longer in D2T patients (*p* = 0.025). The presence of radiographic disease was more frequent in the D2T group (*p* = 0.047). In means of MASES and ASDAS-CRP, higher scores were recorded in the D2T group (both *p* < 0.001). Enthesitis and psoriasis were more frequent in the D2T group (*p* = 0.002 and *p* = 0.006). Regarding comorbidities, hypertension and cardiovascular diseases were more frequent in the D2T group (*p* < 0.001 and *p* = 0.009). The risk of D2T axSpA increased 2.37-fold with the presence of r-axSpA (*p* = 0.018), 1.92-fold with the presence of hypertension (*p* = 0.006), 2.12-fold with the presence of obesity (*p* = 0.024), and 3.63-fold with the presence of psoriasis (*p* = 0.004). Every 1-point increase in MASES increased D2T risk 1.08-fold (*p* = 0.017), and every 1-point increase in ASDAS-CRP increased D2T risk 1.62-fold (*p* < 0.001).

**Conclusions:**

In conclusion, 11.3% of patients with axSpA met the proposed criteria for D2T axSpA. This subgroup was characterized by longer disease duration, higher frequency of r-axSpA, enthesitis, and psoriasis, as well as elevated MASES, CRP, ASDAS-CRP, and BASDAI scores. Hypertension and cardiovascular comorbidities were also significantly more prevalent among D2T patients. These parameters represent potential contributors to treatment complexity and should be carefully considered in therapeutic decision-making. In cases of suboptimal treatment response, reassessment and optimal management of comorbidities are essential, as comorbid conditions can increase disease burden and diminish therapeutic efficacy. Comprehensive care for axSpA should therefore include targeted management of accompanying comorbidities in parallel with disease-specific therapy. Monitoring blood pressure, optimizing body weight, and encouraging smoking cessation are particularly important. Additionally, concomitant rheumatic diseases such as psoriasis, uveitis, or inflammatory bowel disease should be actively evaluated and treated, given their association with more severe disease and reduced treatment response.

**Table:** 

**Key Points** • *Prospectively collected data of 1800 axial spondyloarthritis patients were assessed for this cross-sectional study*. • *11.3% of axial spondyloarthritis patients were identified as difficult-to-treat*. • *Longer disease duration and presence of radiographic axial spondyloarthritis, enthesitis, and psoriasis are more prevalent in difficult-to-treat axial spondyloarthritis patients*. • *Factors and comorbidities complicating axial spondyloarthritis treatment should be considered in treatment plans*.

**Supplementary Information:**

The online version contains supplementary material available at 10.1007/s10067-026-07926-1.

## Introduction

Axial spondyloarthritis (axSpA) is a chronic inflammatory rheumatic disorder primarily involving the axial skeleton and is commonly categorized into two subgroups based on the presence of radiographic sacroiliitis [1]. Patients with sacroiliitis on conventional radiography are categorized as having radiographic axSpA (r-axSpA), whereas those without these radiographic changes are classified as non-radiographic axSpA (nr-axSpA) [2, 3]. Evidence indicates that individuals across these subgroups exhibit comparable clinical manifestations, disease burden, comorbidity profiles, therapeutic requirements, and treatment responses [4, 5]. Given these substantial overlaps, axSpA is increasingly viewed as a single disease spectrum irrespective of radiographic status [6].

Similar to other chronic inflammatory conditions, axSpA markedly impairs health-related quality of life and may contribute to increased mortality [7, 8]. Cardiovascular morbidity and mortality are elevated in axSpA, and heightened inflammatory activity is associated with increased cardiovascular risk. Consequently, early and effective control of disease activity is essential to mitigate the likelihood of cardiovascular events [9, 10].

The treat-to-target strategy has gained prominence in axSpA management [11]. Over the past two decades, the therapeutic landscape of axSpA has expanded considerably with the introduction of targeted agents including tumor necrosis factor inhibitors (TNFi), interleukin-17 inhibitors (IL-17i), and Janus kinase inhibitors (JAKi) [12]. Despite these advancements, remission and low disease activity rates remain suboptimal in routine clinical practice, including at tertiary referral centers [13].

Therapeutic targets cannot be reached for all patients to achieve, and a subset exhibits inadequate response to multiple agents with distinct mechanisms of action. As demonstrated previously in rheumatoid arthritis (RA), such a therapeutic pattern suggests a “difficult-to-treat” (D2T) disease phenotype, now increasingly recognized in axSpA as well [11, 14–17]. The European Alliance of Associations for Rheumatology (EULAR) has defined D2T disease as the persistence of clinically relevant disease activity despite failure of at least two biological or targeted synthetic DMARDs (b/tsDMARDs) with different mechanisms of action [14]. Although initially conceptualized for RA, this framework has been explored as a potential model for defining D2T axSpA, with similar proposals emerging for psoriatic arthritis [15, 18, 19]. One such definition has already been evaluated in a single-center cohort, and most recently, the Assessment of SpondyloArthritis International Society (ASAS) has published a consensus-based expert definition for difficult-to-manage axSpA [18, 20].

As the notion of D2T in axSpA is a novel concept, prevalence and risk factors are not yet well established. Existing reports provide heterogeneous findings, often limited by small sample sizes [12, 16, 17, 21–24]. This large cross-sectional study with 1800 patients from the real-life BioSTaR (Biological and Targeted Synthetic Disease-Modifying Antirheumatic Drugs Registry) database was aimed at determining the prevalence of axSpA patients meeting D2T criteria and at identifying main associated risk factors for D2T axSpA.

The BioSTaR registry, established in 2019 by the Türkiye Romatizma Araştırma ve Savaş Derneği (Turkish League Against Rheumatism, TRASD), is a nationwide digital platform designed to systematically collect data on patients with inflammatory rheumatic diseases receiving b/tsDMARD therapies. It prospectively captures standardized clinical information and patient-reported outcomes, including demographics, disease activity measures, and detailed treatment data, thereby supporting large-scale, multicenter research.

## Materials and methods

### Study design

This multicenter observational cross-sectional study included 1800 patients from the BioSTaR from February 1, 2019, to January 1, 2025. BioSTaR is a national digital registry for inflammatory rheumatic patients receiving b/tsDMARD therapies in Türkiye. The BioSTaR registry routinely and prospectively collects standardized data from rheumatology visits and patient-reported outcomes, including demographics, clinical endpoints, and details regarding antirheumatic medications. For this study, patient data was exported and evaluated as of January 2025. For every patient, all data from their first recorded visit in the database was exported to be analysed.

The local ethical committees approved the study on December 13, 2018 (Türkiye Medicines and Medical Devices Agency, 66,175,679–514.99-E.6366, and Republic of Türkiye Ministry of Health, Ankara Provincial Health Directorate, Ankara Numune Training and Research Hospital, Scientific Studies Ethics Committee, E-18–2413). All procedures were conducted in accordance with the Helsinki Declaration (revision dated 2024). Written informed consent was taken from the participants or from the legally authorized representatives for the participants who were illiterate or vulnerable.

In this multicenter, cross-sectional study, data of all 1800 axSpA patients with b/tsDMARD prescriptions from the BioSTaR (Biological and Targeted Synthetic Disease-Modifying Antirheumatic Drugs Registry) database were classified and analyzed.

### Patients

By January 2025, 1800 axSpA patients were consecutively recorded in the BioSTaR database and all were included for analysis. All patients were > 18 years of age, had been using or were still using at least one b/tsDMARD, and had an axSpA diagnosis according to the ASAS criteria [3].

### Variables

Demographics, including age, sex, body mass index (BMI), marital status, smoking and alcohol use, family history of SpA, and presence of comorbidities, were recorded using a prospective database. The parameters related to SpA, such as disease duration, type of AxSpA (radiographic/non-radiographic), HLA-B27 status, the presence of extra-musculoskeletal manifestations (uveitis, psoriasis (Pso), inflammatory bowel disease (IBD)), arthritis, enthesitis, and dactylitis, were recorded. Comorbidities, including hypertension, cardiovascular disease, diabetes, obesity, and hyperlipidemia, were recorded, and Charlson Index scores were calculated and recorded as well [25]. Maastricht Ankylosing Spondylitis Enthesitis Score (MASES) and disease activity in means of Bath Ankylosing Spondylitis Disease Activity Index (BASDAI) and Ankylosing Spondylitis Disease Activity Score with C-reactive protein (ASDAS-CRP) were recorded [26]. All medication history and currently used medications for axSpA and other diseases were also recorded. For every patient, all data from their first recorded visit in the database were exported to be analysed.

### Outcome measures

The primary objective was to identify the incidence rate of D2T axSpA. The ASAS consensus-based expert definition of D2T axSpA was published after the start of our study [20]. Thus, D2T patients in the BioSTaR database were identified according to the extrapolated definition of D2T axSpA suggested by Wendling et al. [15] (Box 1):

**Box 1** Extrapolated definition of D2T axSpA suggested by Wendling et al. [15]

**Table:** 

All three criteria had to be present for a patient to be classified as D2T axSpA: • Treatment as recommended and failure of ≥ 2 b/tsDMARDs with different mechanisms of action, or 3 b/tsDMARDs • Suggestive evidence of disease activity/progression, defined as ≥ 1 of the following: ⚬ At least moderate activity (ASDAS-CRP > 1.3 or BASDAI > 4/10), ⚬ Signs (including biology and imaging) and/or symptoms suggestive of active disease (articular or extra rheumatological: uveitis, Pso, IBD), ⚬ Inability to reduce or discontinue nonsteroidal anti-inflammatory drugs (NSAIDs), ⚬ Disease controlled, but with persistent axSpA symptoms causing reduced quality of life • Management of signs and/or symptoms is perceived as problematic by the rheumatologist and/or patient	

ASDAS-CRP: ankylosing spondylitis disease activity score with C-reactive protein; axSpA: axial spondyloarthritis; BASDAI: Bath Ankylosing Spondylitis Disease Activity Index; b/tsDMARDs: biological/targeted synthetic disease-modifying antirheumatic drugs; D2T: difficult to treat; IBD: inflammatory bowel disease; nr-axSpA: radiographic axial spondyloarthritis; Pso: psoriasis; r-axSpA: non-radiographic axial spondyloarthritis

The secondary objective was to identify the main associated factors for D2T axSpA.

### Statistical analysis

The Shapiro–Wilk test was used for assessing whether the variables follow normal distribution or not. Continuous variables were presented as median (IQR) values. Categorical variables were reported as *n* (%). According to the normality test results, the Mann–Whitney *U* test was used for comparisons between two independent groups; Wilcoxon test was used for comparisons between two dependent groups. Categorical variables were compared between the groups using the Pearson Chi-square test, Fisher’s Exact test, and Fisher–Freeman–Halton test. Correlations between continuous variables were examined by correlation analysis, and Spearman’s correlation coefficients were calculated. Jamovi (Version 2.6. Released 2025) was used for statistical analysis, and a *p* value < 0.05 was considered statistically significant.

## Results

**Fig. 1:**
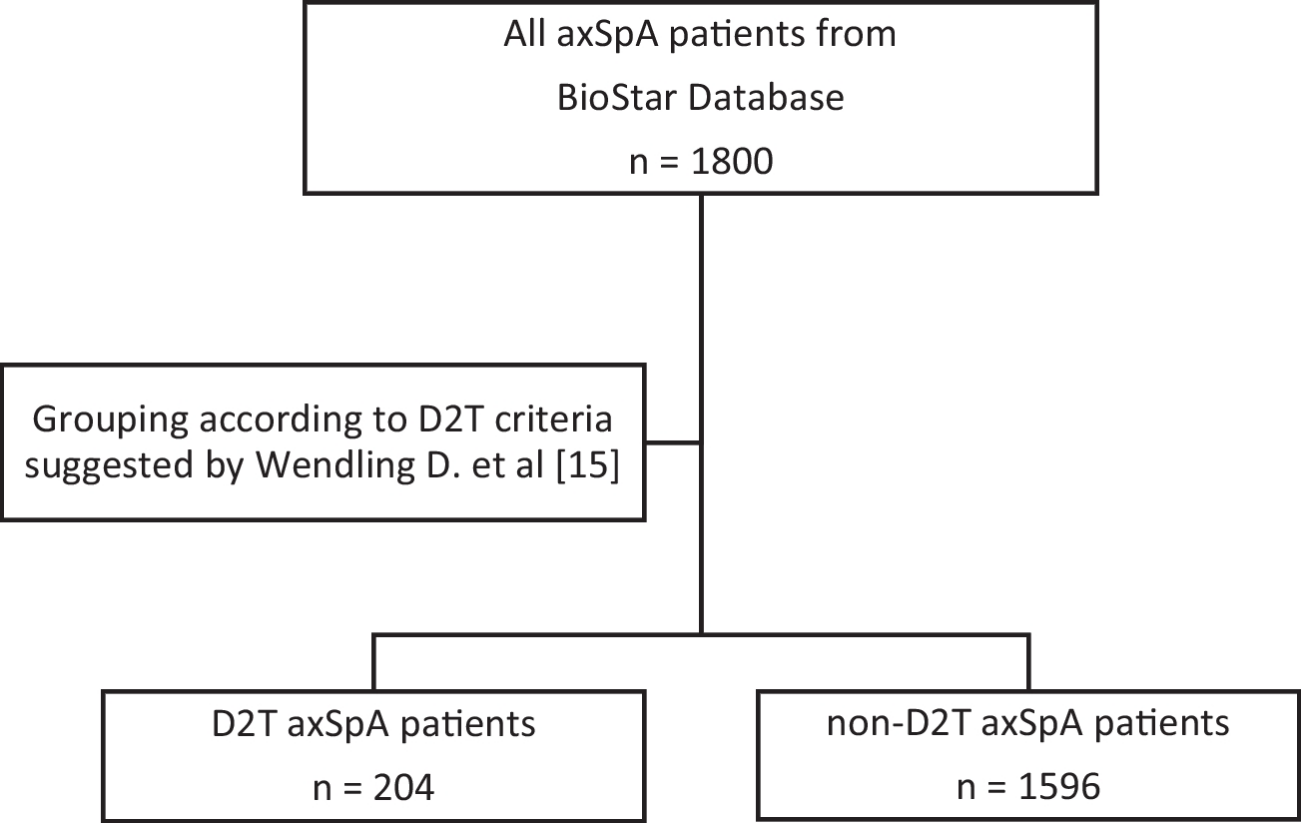
Flow chart of study patient groups. axSpA: axial spondyloarthritis; BioSTaR: Biological and Targeted Synthetic Disease-Modifying Antirheumatic Drugs Registry; b/tsDMARDs: biological/targeted synthetic disease-modifying antirheumatic drugs; D2T: difficult to treat

According to the criteria mentioned above, of the 1800 axSpA patients recorded in the BioSTaR database, 204 were classified as D2T axSpA (Fig. 1). Data of these patients were compared to the data from 1596 non-D2T axSpA patients.

**Fig. 2:**
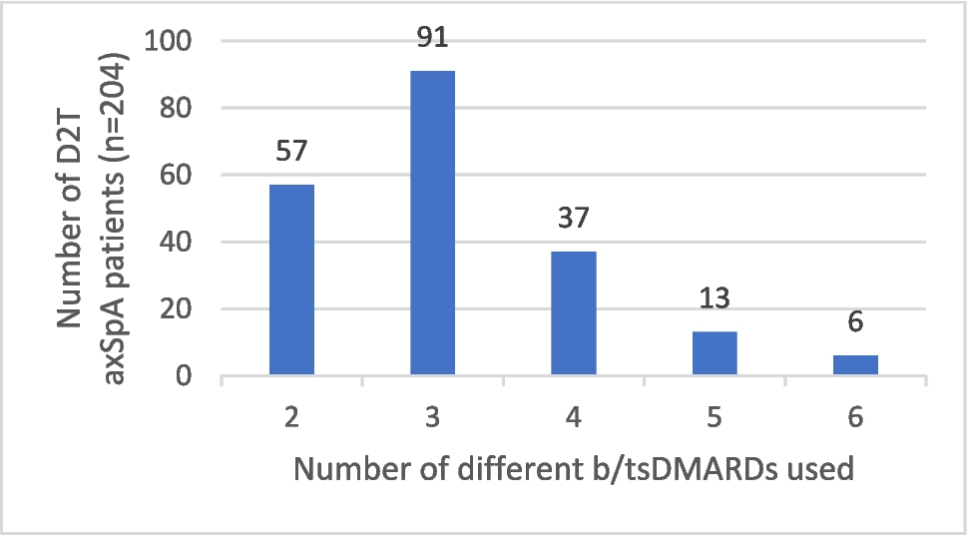
Number of D2T axSpA patients using different numbers of b/tsDMARDs. axSpA: axial spondyloarthritis; b/tsDMARDs: biological/targeted synthetic disease-modifying antirheumatic drugs; D2T: difficult to treat

**Fig. 3:**
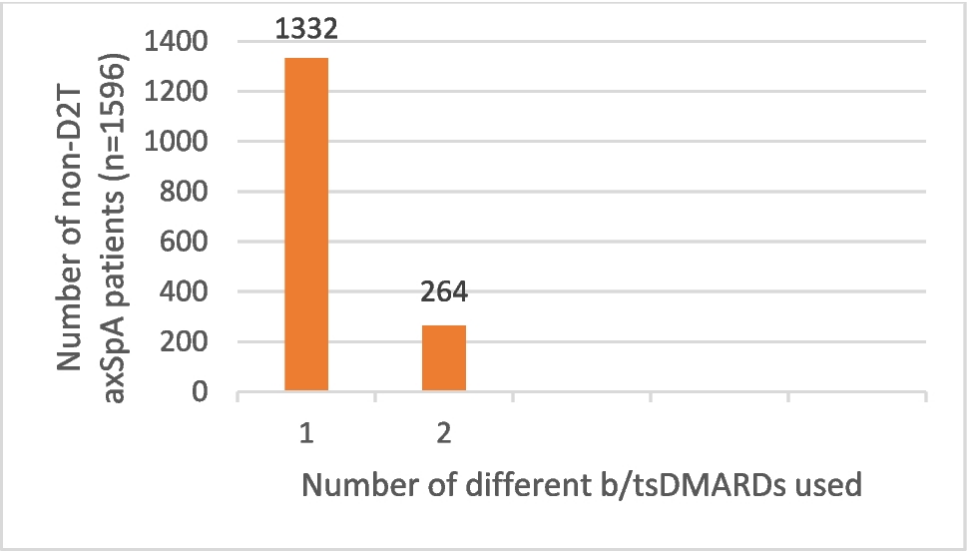
Number of non-D2T axSpA patients using different numbers of b/tsDMARDs. axSpA: axial spondyloarthritis; b/tsDMARDs: biological/targeted synthetic disease-modifying antirheumatic drugs; D2T: difficult to treat

After the separation into D2T and non-D2T axSpA groups, distributions of patients according to the number of previously used b/tsDMARDs are presented in Figs. 2 and 3.

Currently or previously used all b/tsDMARD treatments by patients in the database are listed in Table 1. Treatments with earlier authorization dates were used more often; hence, adalimumab followed by etanercept was the most prescribed b/tsDMARD treatment in both groups. As the authorization of JAKi in the treatment of axSpA was much more recent in Türkiye, it was prescribed the least.

**Table 1:** Currently or previously used all b/tsDMARD treatments by patients with D2T axSpA and non-D2T axSpA

Used b/tsDMARD treatments	D2T axSpA (204)	nD2T axSpA (1596)
Adalimumab	141 (69.1)	666 (41.7)
Certolizumab	51 (25)	142 (8.9)
Etanercept	111 (54.4)	542 (34)
Golimumab	81 (39.7)	400 (25.1)
Infliximab	59 (28.9)	227 (14.2)
JAKi	3 (1.5)	10 (0.6)
Secukinumab	94 (46.1)	138 (8.7)

*axSpA* axial spondyloarthritis, *b/tsDMARDs* biological/targeted synthetic disease-modifying antirheumatic drugs, *D2T* difficult to treat

Comparison of sociodemographic characteristics, extra-musculoskeletal manifestations, comorbidities, and previous b/tsDMARD treatments between patients with D2T axSpA and non-D2T axSpA was made with bivariate analysis (Table 2).

**Table 2:** Bivariate analysis and comparison of sociodemographic characteristics, extra-musculoskeletal manifestations, and comorbidities between patients with D2T axSpA and non-D2T axSpA

	***n***	**D2T axSpA**	***n***	**non-D2T axSpA**	***p*-value**
**Sociodemographics**					
Female Sex	204	73 (35.8)	1596	510 (32)	0.271^a^
Age, median (IQR)	204	46 (12.5)	1596	45 (15)	0.657^b^
axSpA duration (months), median (IQR)	204	162 (151)	1596	148 (149)	**0.025**^**b**^
Marital status	204		1596		
• Married		167 (81.9)		1302 (81.6)	0.922^a^
• Single		37 (18.1)		294 (18.4)	
Having children	204	151 (74)	1596	1234 (77.3)	0.292^a^
BMI, median (IQR)	204	27.34 (6.5)	1596	26.76 (5.4)	0.206^b^
Smoking status	198		1501		
• Never		77 (38.9)		668 (44.5)	0.187^a^
• Past		31 (15.7)		251 (16.7)	
• Current		90 (45.5)		582 (38.8)	
Alcohol status	192		1427		
• Never		164 (85.4)		1222 (85.6)	0.852^a^
• Past		6 (3.1)		54 (3.8)	
• Current		22 (11.5)		151 (10.6)	
**axSpA clinical features**					
Radiographic status	204		1596		
• nr-axSpA		14 (6.9)		184 (11.5)	**0.047**^**a**^
• r-axSpA		190 (93.1)		1412 (88.5)	
Arthritis	88	26 (29.5)	715	154 (21.5)	0.089^a^
Dactylitis	204	6 (2.9)	1596	43 (2.7)	0.838^a^
Enthesitis	155	107 (69)	1130	630 (55.8)	**0.002**^**a**^
MASES	155	2 (6)	1101	1 (3)	** < 0.001**^**b**^
ASDAS-CRP	200	2.7 (1.2)	1552	2.2 (1.3)	** < 0.001**^**b**^
HLA-B27 positivity	155	47 (30.3)	1090	396 (36.3)	0.162^a^
CRP (mg/L), median (IQR)	201	5 (10.7)	1550	3 (6.4)	** < 0.001**^**b**^
BASDAI, median (IQR)	200	3.8 (3.1)	1500	3 (2.7)	** < 0.001**^**b**^
**Extra-musculoskeletal manifestations and findings**					
Pso	204	9 (4.4)	1595	22 (1.4)	**0.006**^**d**^
Uveitis	204	31 (15.2)	1596	196 (12.3)	0.238^a^
IBD	204	6 (2.9)	1596	36 (2.3)	0.466^d^
**Comorbidities**					
Diabetes mellitus	198	13 (6.6)	1392	109 (7.8)	0.532^a^
Hyperlipidemia	96	11 (11.5)	703	68 (9.7)	0.582^a^
Hypertension	197	50 (25.4)	1398	201 (14.4)	** < 0.001**^**a**^
Cardiovascular disease	183	14 (7.7)	1421	51 (3.6)	**0.009**^**a**^
Obesity	204	25 (12.3)	1596	203 (12.7)	0.851^a^
Charlson score = 0	204	103 (50.5)	1596	851 (53.3)	0.740^a^
Charlson score = 1		57 (27.9)		426 (26.7)	
Charlson score ≥ 2		44 (21.6)		319 (20)	

Data was presented as *n* (%) and median(IQR) unless otherwise stated

*ASDAS-CRP* ankylosing spondylitis disease activity score with C-reactive protein, *axSpA* axial spondyloarthritis, *BASDAI* Bath Ankylosing Spondylitis Disease Activity Index, *BMI* Body mass index, *b/tsDMARDs* biological/targeted synthetic disease-modifying antirheumatic drugs, *CRP* C-reactive protein, *D2T* difficult to treat, *HLA* human leukocyte antigen, IBD inflammatory bowel disease, *IQR* interquartile range, *MASES* Maastricht Ankylosing Spondylitis Enthesitis Score, *nr-axSpA* radiographic axial spondyloarthritis, *Pso* psoriasis, *r-axSpA* non-radiographic axial spondyloarthritis

^a^Chi-square test. ^b^Mann–Whitney *U* test. ^c^Fisher–Freeman–Halton test. ^d^Fisher’s exact test

In this analysis, the only difference found between D2T and non-D2T axSpA groups in means of sociodemographic characteristics was axSpA disease duration, which was longer in D2T patients (*p* = 0.025). There were also significant differences in axSpA clinical features, extra-musculoskeletal manifestations, and comorbidities. The presence of r-axSpA patients was more frequent in the D2T group (*p* = 0.047). In means of MASES and ASDAS-CRP, higher scores were recorded in the D2T group (both *p* < 0.001). Enthesitis and Pso were more frequent in the D2T group (*p* = 0.002 and *p* = 0.006). Regarding comorbidities, hypertension and cardiovascular diseases were more frequent in the D2T group (*p* < 0.001 and *p* = 0.009).

Parameters in the bivariate analysis were also evaluated by multivariate analysis to identify risk factors for D2T axSpA (Table 3). In the multivariate logistic regression analysis, variable selection was performed using the forward elimination method and the resulting logistic regression model was found to be significant (*p* < 0.001). Risk of D2T axSpA increased 2.37-fold with the presence of r-axSpA (*p* = 0.018), 1.92-fold with the presence of hypertension (*p* = 0.006), 2.12-fold with the presence of obesity (*p* = 0.024), and 3.63-fold with the presence of Pso (*p* = 0.004). Every 1-point increase in MASES increased D2T risk 1.08-fold (*p* = 0.017), and every 1-point increase ASDAS-CRP increased D2T risk 1.62-fold (*p* < 0.001).

**Table 3:** Multivariate analysis of factors associated with D2T axSpA

	**OR (95% CI)**	***p*-value**
r-axSpA	2.37 [1.16 to 4.86]	**0.018**
Hypertension	1.92 [1.21 to 3.05]	**0.006**
Obesity	2.12 [1.11 to 4.07]	**0.024**
Pso	3.63 [1.50 to 8.81]	**0.004**
MASES	1.08 [1.01 to 1.14]	**0.017**
ASDAS-CRP	1.62 [1.32 to 2]	** < 0.001**

*ASDAS-CRP* ankylosing spondylitis disease activity score with C-reactive protein, *MASES* Maastricht Ankylosing Spondylitis Enthesitis Score, *OR* odds ratio, *Pso* psoriasis, *r-axSpA* non-radiographic axial spondyloarthritis

In this study, logistic regression analysis was conducted to determine the risk factors affecting the occurrence of D2T in axSpA patients. A forward selection approach was adopted as the variable selection method. The analysis results showed that the logistic regression model obtained in the final step was compatible with the data (Hosmer and Lemeshow test *p* = 0.769; *R*^2^ = 0.128), and the resulting logistic regression model was also significant (*p* < 0.001).

## Discussion

In this study, all 1800 axSpA patients from the BioSTaR database were classified and analyzed for D2T disease rate and main associated factors for D2T axSpA. Of these 1800 axSpA patients, 204 (11.3%) were identified as D2T axSpA. In a study investigating 10.798 axSpA patients from a national administrative database from France, 19.6% were defined as D2T [11]. In another study with 311 axSpA patients from 3 centers from northern France by Philippoteaux et al. D2T axSpA ratio was reported as high as 28.3% [16]. In another recent cross-sectional study from Türkiye by Öğüt et al., 166 consecutive axSpA patients were evaluated and 38 patients (22.9%) fulfilled D2T criteria [17].

Following the publication of the ASAS consensus-based expert definition for difficult-to-manage (D2M) axSpA, an increasing number of studies adopted and applied this framework. A single-center cross-sectional study from Portugal by Abreu et al. analyzed 207 axSpA patients and reported 2.9% had D2M axSpA according to ASAS criteria [21]. Another single-center cohort study with 129 axSpA patients from Argentina by Garcia-Salinas et al. reported 8.5% of D2M disease [22]. Lee et al. reported only 1.7% D2M axSpA from the KOBIO registry of Korea, but most of the patient population was b/tsDMARD naïve (1784/2541) [23]. In a cross-sectional study with 263 patients from the Netherlands, Smiths et al. reported 9.7% of axSpA patients had D2M disease [24]. The ratio of D2T patients from our study is in the range of these previous results of axSpA studies. As BioSTaR is a novel, broad, and living database, new data will be reanalyzed in upcoming years, which might change this ratio.

In terms of sociodemographic characteristics, the D2T group had a significantly longer disease duration than the non-D2T group in concordance with these previous studies. In these studies, female gender was more prominent in D2T groups, but there was no difference between genders in our study, in contrast to their results [12, 16, 17].

In our study, data on axSpA features showed that the D2T axSpA group had significantly more r-axSpA patients compared to nr-axSpA patients (93.1% vs 88.5%, *p* = 0.047). Multivariate logistic regression analysis showed that the risk of D2T axSpA increased 2.37-fold with the presence of r-axSpA (*p* = 0.018). Philippoteaux et al. also reported that a higher ratio of r-axSpA patients was in the D2T group, but overall r-axSpA prevalence was much higher in our study in comparison to this study where the D2T group had 71.2% r-axSpA patients and the non-D2T group had 60.3% [16]. In the studies from Portugal by Abreu et al. and Korea by Lee K.A. et al., r-axSpA ratios were high and comparable to our results (84.5% and 90.6%, respectively) [27, 28]. In the other study from Türkiye, there weren’t any differences between groups (86.8% vs 85.2%), but the higher r-axSpA prevalence was similar to our findings [17].

A meta-analysis reviewing peripheral manifestations in axSpA showed the pooled prevalences of peripheral arthritis as 29.7%, enthesitis as 28.8%, and dactylitis as 6.0% in patients with r-axSpA, and similar prevalences were reported in patients in nr-axSpA [29]. It is well known that some of these peripheral manifestations might influence treatment decisions [30]. In our study, in the D2T group, significantly more patients had enthesitis (*p* = 0.002), but there were no significant differences in means of arthritis or dactylitis. Identifying enthesitis is crucial both for differentiating among inflammatory rheumatic diseases and for managing appropriate treatment. The introduction of novel therapeutic agents may demonstrate efficacy on enthesis that might update current treatment strategies for axSpA.

As expected from a higher rate of enthesitis in our study, MASES was significantly higher in the D2T group as well (*p* < 0.001), and every point of increase in MASES slightly increased the risk for D2T disease (*p* = 0.017). In the study by Philippoteaux et al., there were no differences in enthesitis or dactylitis rates, but peripheral spondyloarthritis was higher in the D2T group (34.9% vs 21.4%, *p* < 0.05) [16]. In our study, we assessed disease severity with ASDAS-CRP, and it was higher in the D2T group as expected (2.7 vs 2.2, *p* < 0.001), and this result is similar to the result from the earlier cross-sectional study from Türkiye (3.1 vs 1.8, *p* < 0.05) [17]. And every 1-point increase in ASDAS-CRP increased D2T risk 1.62-fold (*p* < 0.001). BASDAI was significantly higher in the D2T group (3.8 vs 3, *p* < 0.001). In the studies by Philippoteaux et al. and Öğüt et al., BASDAI was also higher in the D2T groups (*p* < 0.05 and *p* < 0.001) [16, 17].

Extra-musculoskeletal manifestations can frequently accompany axSpA and might be indicators for disease severity [8, 11, 31–34]. Results from a meta-analysis reported the prevalence of Pso as 9.3%, uveitis as 25.8%, and IBD as 6.8% in patients with r-axSpA [33]. In a population-based study, IBD was associated with higher disease activity, and Pso was associated with both higher disease activity and functional impairment [34]. In this study, the presence of Pso, uveitis, and IBD was analyzed in D2T and non-D2T groups. Even though all were more frequent in the D2T group, only the Pso rate was significantly higher (4.4% vs 1.4%, *p* = 0.006). Multivariate analysis indicated a 3.63-fold increase in the risk of D2T disease with the presence of Pso (*p* = 0.004). In the French national database study, accompanying Pso was also significantly higher in the D2T group (49% vs 37.4%, *p* < 0.001) [11]. Similarly, in the study by Philippoteaux et al., Pso was also significantly higher in the D2T group (21% vs 20.7%, *p* < 0.05) [16].

In axSpA, comorbidities are frequent and are associated with higher disease activity and increased functional impairment [31, 35]. In a recent meta-analysis, the most prevalent comorbidities were reported as hypertension (23%), hyperlipidemia (17%), and obesity (14%), and the increased burden of comorbidities was associated with higher disease severity and increased mortality [27]. In a cross-sectional study from the BioSTaR registry involving 1242 axSpA patients, the most common comorbidities were hypertension (13.4%) and diabetes mellitus (6.7%) [32]. In our current study, the most common comorbidities were similar, and some were more common in the D2T group. Hypertension and cardiovascular disease were significantly more common in D2T patients (*p* < 0.001 and *p* = 0.009). Hypertension was also calculated to be a risk factor for D2T axSpA; with the presence of hypertension, D2T disease risk increased 1.92-fold (*p* = 0.006). Even though obesity rates in the D2T and non-D2T groups were not different, obesity was calculated to be a risk factor in multivariate analysis with a 2.12-fold increase for D2T disease (*p* = 0.024). The link between these comorbidities and suboptimal therapeutic outcomes appears to be multifactorial, involving both shared inflammatory mechanisms and independent pathways that impair treatment response. Obesity is increasingly recognized as a chronic low-grade inflammatory state characterized by excessive production of adipokines and proinflammatory cytokines. These mediators overlap substantially with the cytokine networks implicated in axSpA pathogenesis. Adipose tissue-derived inflammation may therefore amplify baseline disease activity, sustain systemic inflammation, and exacerbate musculoskeletal symptoms. In parallel, hypertension has been linked to endothelial dysfunction and vascular inflammation—a process strongly driven by TNF-α, IL-17, and oxidative stress—which may compound the systemic inflammatory setting already present in axSpA. Thus, both obesity and hypertension may biologically reinforce inflammatory circuits central to axSpA, creating a physiological context in which disease becomes harder to control [28, 36–38].

In Türkiye, JAKi’s and IL-17i’s received regulatory approval for the treatment of axSpA considerably later than other b/tsDMARDs, resulting in lower prescription and utilization rates. Earlier access to and more rapid transition between therapeutic agents with distinct mechanisms of action may help reduce the prevalence of D2T disease in the future. Given their potential efficacy in musculoskeletal domains, as well as extra-musculoskeletal conditions including Pso, uveitis, and IBD, the integration of these newer therapies has the potential to further enhance treatment outcomes in axSpA [6].

The main limitation of our study was the unavailability of an established D2T axSpA definition at the initiation of this study. The only suggested D2T axSpA criteria was from Wendling et al. Thus, we used it to classify D2T and non-D2T patients [15]. ASAS consensus-based expert definition of D2M axSpA was published after the beginning of our data export and analysis process for this study [20]. But it should be kept in mind that the difference between ASAS D2M definition and D2T definition by Wendling et al. is minor (Supplementary Table). Two of these differences might have affected our results. Criteria from Wendling et al. includes “…treatment as recommended and failure of ≥ 2 b/tsDMARDs with different mechanisms of action, or 3 b/tsDMARDs” where ASAS criteria include only “…failure of ≥ 2 b/tsDMARDs with different mechanisms of action”. As stated before, novel biologic or targeted drug use is lower in Türkiye as a result of late authorizations. As a result, different medications with the same mechanism of action can be prescribed more often. This might have caused more patients to be in the D2T group since Wendling et al. criteria include the use and failure of 3 b/tsDMARDs. The other main criteria difference is in disease severity. Wendling et al. criteria include “At least moderate activity (ASDAS-CRP > 1.3 or BASDAI > 4/10)” where ASAS criteria include “High or very high disease activity (ASDAS ≥ 2.1).” This might have caused less severe patients to be granted as D2T in our study. In our following axSpA analysis and publications, ASAS consensus-based expert definition of D2M axSpA will be used and compared with these previous results. In addition, as this study’s data analysis was made from a single time point for each patient, no follow-up data was analyzed. In future studies, follow-up data will be used for longitudinal assessment giving us more information on change in D2T status, drug persistence and switches.

As BioSTaR is a nationwide database, complete standardization of imaging interpretation was not possible as every center had different radiologists, and MRI was regarded as redundant in cases where sacroiliitis was already present in X-rays. However, definitions of “ASAS MRI working group” were implemented in every center to maximize imaging standardization [39].

Even though there is a large pool of studies regarding D2T RA, there are only a few on the subject of D2T axSpA. As a result, our study stands out as it analyzes a large group of prospectively collected patient data. Even though the data was collected prospectively, some of the variables had missing data (Table 2). These missing data were random but might still have created a bias.

In conclusion, 11.3% of patients with axSpA met the proposed criteria for D2T axSpA. This subgroup was characterized by longer disease duration, higher frequency of r-axSpA, enthesitis, and Pso, as well as elevated MASES, CRP, ASDAS-CRP, and BASDAI scores. Hypertension and cardiovascular comorbidities were also significantly more prevalent among D2T patients. These parameters represent potential contributors to treatment complexity and should be carefully considered in therapeutic decision-making. In cases of suboptimal treatment response, reassessment and optimal management of comorbidities are essential, as comorbid conditions can increase disease burden and diminish therapeutic efficacy. Comprehensive care for axSpA should therefore include targeted management of accompanying comorbidities in parallel with disease-specific therapy. Monitoring blood pressure, optimizing body weight, and encouraging smoking cessation are particularly important. Additionally, concomitant rheumatic diseases such as Pso, uveitis, or IBD should be actively evaluated and treated, given their association with more severe disease and reduced treatment response [6].

## Supplementary Information

Below is the link to the electronic supplementary material.

ESM 1 (DOCX 12.9 KB)

## Data Availability

The data that supports the findings of this study is available on request from the corresponding author. The data is not publicly available due to patient privacy.
