# Supplementary material for: Prevalence and risk factors of difficult-to-treat axial spondyloarthritis: Real-life evidence from the BioSTaR database
Source: Clin Rheumatol. 2026 Jan 19;45(2):945–56. doi: 10.1007/s10067-026-07926-1 (PMC12858463; doi:10.1007/s10067-026-07926-1)
Supplement: Supplementary file 1 — (DOCX 12.9 KB) [file 10067_2026_7926_MOESM1_ESM.docx]

**Supplementary Table:**

Extrapolated definition of D2T axSpA suggested by Wendling D. et al [15] and consensus-based expert definition of difficult-to-manage axSpA by ASAS [20] for side-by-side comparison.

| • Treatment as recommended and failure of ≥2 b/tsDMARDs with different mechanisms of action, or 3 b/tsDMARDs.  • Suggestive evidence of disease activity/progression, defined as ≥1 of the following:  o At least moderate activity (ASDAS-CRP >1.3 or BASDAI >4/10),  o Signs (including biology and imaging) and/or symptoms suggestive of active disease (articular or extra rheumatological: uveitis, Pso, IBD),  o Inability to reduce or discontinue nonsteroidal anti-inflammatory drugs (NSAIDs),  o Disease controlled, but with persistent axSpA symptoms causing reduced quality of life.  • Management of signs and/or symptoms is perceived as problematic by the rheumatologist and/or patient.  All 3 criteria must be present |
| --- |
| All three criteria must be present in a patient with axSpA diagnosed by a rheumatologist:  1. Treatment according to the ASAS-EULAR recommendations and failure of ≥2 b/tsDMARDs* with different mechanisms of action (unless contraindicated**).  2. Insufficient control of signs/symptoms of axSpA defined as ≥1 of the following:  a. High or very high disease activity (ASDAS ≥2.1);  b. Signs or symptoms suggestive of active disease (musculoskeletal or extra-musculoskeletal manifestations, elevated CRP***, active inflammation on MRI***);  c. Rapid radiographic spinal progression****;  d. Well-controlled disease according to the above-mentioned points (a-c), but still having axSpA symptoms that are causing a reduction in QoL.  3. The present signs/symptoms are percieved as problematic by the rheumatologist and/or the patient.  *Including primary and secondary failure, or discontinuation because of side effects/intolerability/contraindications. Treatment failure but not discontinuation due to side effects/intolerability/contraindications is mandatory to conclude about the presence of treatment-refractory disease.  **Contraindications, which result in the inability to apply at least 2 b/tsDMARDs.  ***Objective signs of inflammatory activity (elevated CRP or active inflammation on MRI of sacroiliac joints or spine) are mandatory to conclue about the presence of treatment-refractory disease.  ****Defined as development of >2 new syndesmophytes/bony bridges in 2 years. |
